# Red cell distribution width-to-albumin ratio and hypertension risk: age-specific threshold effects identified in the 2017–2020 NHANES U.S. Adult population

**DOI:** 10.1186/s12872-025-05072-1

**Published:** 2025-08-09

**Authors:** Zihao Zhao, Yuhong Ma, Weizhong Huangfu

**Affiliations:** Department of General Practice, The Affiliated Hospital of Inner Mongolia Medical University, Hohhot, 010000 Inner Mongolia Autonomous Region China

**Keywords:** Red cell distribution width-to-albumin ratio, Hypertension, Complex sampling weighting, NHANES

## Abstract

**Background:**

As a major modifiable risk factor for cardiovascular diseases worldwide, hypertension novel biomarkers that integrate inflammatory and metabolic pathways may improve risk stratification. The association between the red cell distribution width-to-albumin ratio (RAR), a newly identified inflammatory biomarker, and hypertension has not been systematically evaluated in population-based studies.

**Methods:**

A cross-sectional study included 7,878 adults. Weighted multivariable logistic regression and threshold effect models were employed to analyze nonlinear associations, with subgroup analyses exploring heterogeneity.

**Results:**

RAR showed a linear positive association with hypertension (adjusted OR = 1.26 per unit, 95%CI:1.09–1.44, *P* < 0.05), a threshold effect was observed. Piecewise regression revealed a significant association when RAR ≥ 3.4, with a higher hypertension prevalence (adjusted OR = 1.34, 95%CI:1.17–1.54), while no association existed below 3.4 (*P* = 0.408). For those aged 40–60 years, the inflection point was RAR = 3.92 (95%CI:3.76–4.51), with stronger associations observed below this threshold (OR = 1.80, 95%CI:1.33–2.43). Subgroup analyses revealed significant heterogeneity: diabetics exhibited stronger associations than non-diabetics (interaction *P* = 0.02), and enhanced associations were also observed in females and individuals aged > 40 years.

**Conclusion:**

This study confirmed a linear positive correlation between red blood cell distribution width and albumin ratio (RAR) and the prevalence of hypertension, RAR ≥ 3.4 was associated with higher hypertension prevalence and may help identify high-risk subgroups, particularly among diabetics, but its predictive value warrants validation through prospective cohort studies.

## Introduction

Hypertension, a major modifiable risk factor for cardiovascular disease (CVD), affects over 1.3 billion adults globally and substantially contributes to morbidity and mortality from stroke, myocardial infarction, and renal failure [1–3]. Despite advances in diagnostic and therapeutic strategies, nearly half of hypertensive patients exhibit inadequate blood pressure control, underscoring the need for novel biomarkers to improve risk stratification and elucidate underlying pathophysiological mechanisms [4]. Chronic inflammation, oxidative stress, and endothelial dysfunction synergistically drive hypertension pathogenesis. Among them, Inflammatory factors (e.g., TNF-α and IL-6) can activate the renin-angiotensin system, promoting vascular smooth muscle cell proliferation. Oxidative stress damages vascular endothelial cells, reduces nitric oxide (NO) bioavailability, and impairs vascular dilation. Endothelial dysfunction further exacerbates hemodynamic abnormalities, forming a vicious cycle [5]. 

The red cell distribution width-to-albumin ratio (RAR) is an emerging composite biomarker [6]. An elevated red cell distribution width (RDW) has traditionally been used for the assessment of anemia [7]. However, recent studies have found that it is independently associated with adverse cardiovascular outcomes [8], which may be related to disorders of erythropoiesis, subclinical inflammation, and oxidative damage [9, 10]. Hypoalbuminemia, on the other hand, indicates systemic inflammation, endothelial dysfunction, and malnutrition [11, 12]. As an integrator of inflammation-oxidative stress-vascular injury pathways, RAR may reflect pathways contributing to vascular dysfunction in hypertension development.

The red cell distribution width-to-albumin ratio has been proven to be associated with a variety of inflammatory diseases [13], including diabetes mellitus and its microvascular complications [14], autoimmune diseases such as rheumatoid arthritis [15, 16], critical illnesses such as sepsis [17, 18], cerebrovascular events [19], and cardiovascular diseases such as heart failure [20, 21].Recent studies have explored the prognostic value of RAR in critical illnesses and postoperative complications [19, 22], epidemiological evidence linking RAR to hypertension through these mechanistic pathways remains lacking. The lack of studies on the association threshold of RAR with hypertension based on the general population.

Therefore, this study utilized data from the National Health and Nutrition Examination Survey (NHANES) 2017–2020 to achieve the following specific objectives: To evaluate the cross-sectional association between RAR and hypertension in the U.S. adult population. To explore heterogeneity in the association between RAR and hypertension through subgroup analysis: Stratify participants by age (< 60 years/≥60 years), gender, BMI (< 25/25–30/≥30 kg/m²), race, and diabetes status. To analyze between-group differences in the strength of association between RAR and hypertension, and assess the statistical significance of subgroup effects using interaction tests.

## Methods

### Study design and population

The data utilized in this study were derived from the 2017–2020 cycles of the National Health and Nutrition Examination Survey (NHANES), an ongoing, cross-sectional surveillance program conducted by the Centers for Disease Control and Prevention (CDC). NHANES employed a complex, multistage, stratified probability sampling design to select a nationally representative sample of non-institutionalized U.S. residents, with the primary objective of assessing population health trends and nutritional status across all age groups. The study protocol received ethical approval from the National Center for Health Statistics (NCHS) Research Ethics Review Board. Written informed consent was obtained from all adult participants and legally authorized representatives of minors prior to data collection. Comprehensive documentation regarding the survey methodology and ethical considerations is accessible through the official NHANES website at www.cdc.gov/nchs/nhanes/irba98.htm.

**Fig. 1:**
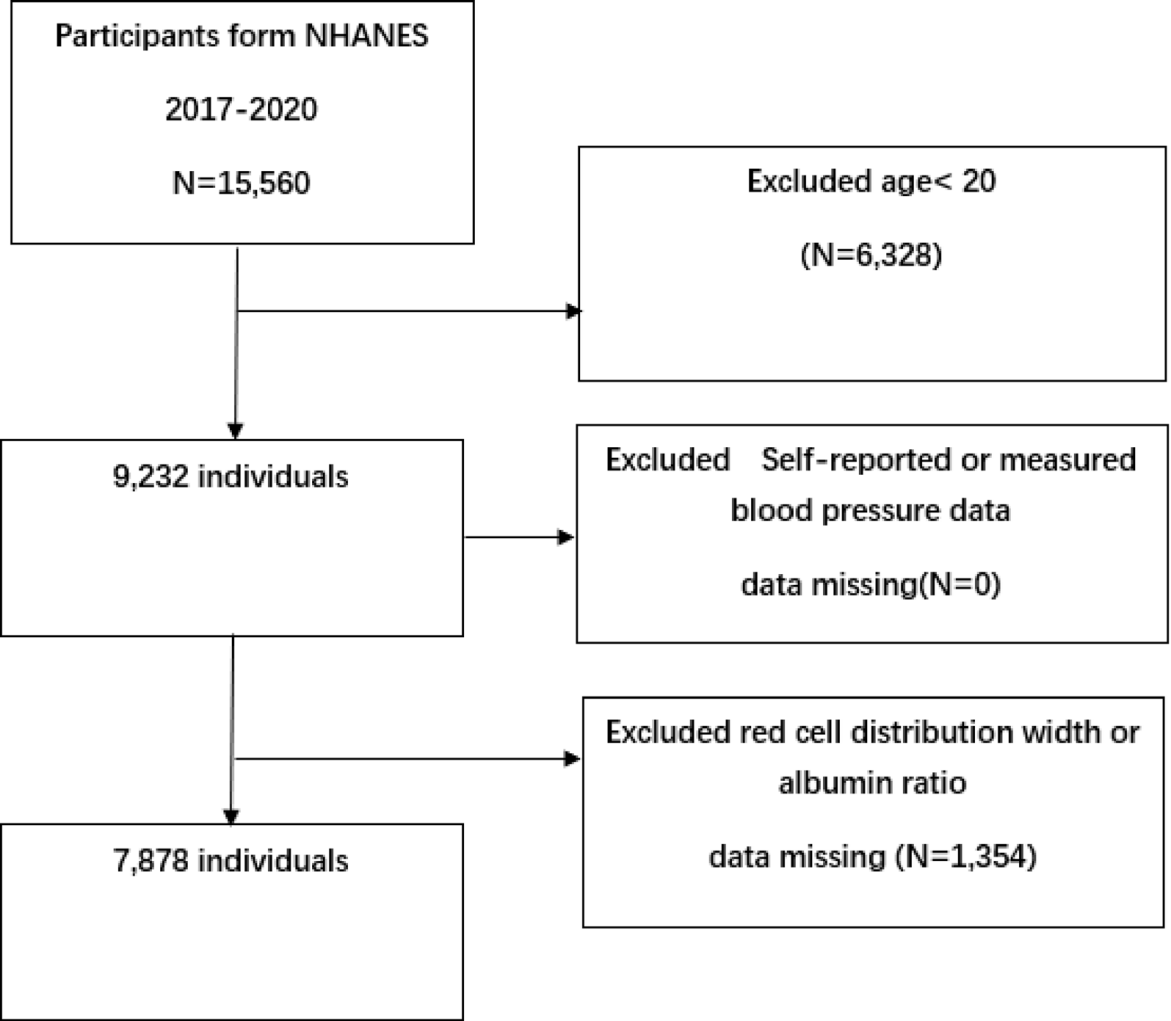
Sample Selection Flowchart of NHANES 2017–2020. Flowchart of participant selection

This cross-sectional study utilized data from the National Health and Nutrition Examination Survey (NHANES) conducted from 2017 to 2020, covering an initial total of 15,560 participants. Exclusion criteria included: (1) individuals under the age of 20 (a total of 6,328); and (2) individuals with missing self-reported hypertension or blood pressure measurement data (0 individuals) and (3) individuals with missing RAR data (1,354 individuals). After these exclusions, the final analysis sample group consisted of 7,878 eligible participants (Fig. 1).

### Exposure and outcome variables

Red cell distribution width/albumin ratio (RAR) was calculated as follows: RAR = Red cell distribution width (%) / serum albumin (g/dL) [23]. The data on red blood cell distribution width was derived from the complete blood cell count data in the NHANES database from 2017 to 2020, while the serum albumin data was obtained from the standard biochemical profile data in the same database from 2017 to 2020. For specific measurement methods, please refer to the NHANES Laboratory/Medical Technicians Procedure Manual.

Hypertension.

The determination of hypertension cases was made through the following methods. (1) Participants who answered “Yes” to the question “Has a doctor or health professional ever told you that you have high blood pressure?” were classified as having hypertension. This diagnostic classification is consistent with the standards established in the Seventh Report of the Joint National Committee on Prevention, Detection, Evaluation, and Treatment of High Blood Pressure (JNC 7) [24]. (2) The average measured systolic blood pressure was > 140 mmHg or the average diastolic blood pressure was > 90 mmHg. (3) Those receiving oral antihypertensive drug treatment. Ultimately, a total of 7,878 patients were included in the study.

Covariates.

The adjusted covariates encompassed multidimensional parameters: (1) Demographic characteristics: sex, age (continuous), race/ethnicity; (2) Socioeconomic status: family poverty-to-income ratio (PIR), educational attainment, marital status; (3) Lifestyle factors: alcohol consumption status, smoking exposure; (4) Comorbidities: self-reported history of hypercholesterolemia, diabetes mellitus, and nephrolithiasis. These variables were systematically collected through structured questionnaires and physical examinations in the NHANES protocol. These covariates were selected to comprehensively account for potential confounders and ensure robust analysis of the association between RAR and hypertension.

### Statistical analysis

We conducted statistical analyses using R software (version 4.5.1; R Foundation for Statistical Computing), incorporating the NHANES complex sampling weights to account for the stratified multistage probability sampling design. Statistical significance was set at a two-sided alpha level of 0.05. Participants were categorized into four groups based on quartiles of the RDW to albumin ratio (RAR): Q1 (*n* = 1961), Q2 (*n* = 1947), Q3 (*n* = 1994), and Q4 (*n* = 1976). Continuous variables were compared using complex survey design-adjusted Student’s t-tests, and categorical variables were compared using complex survey design-adjusted Pearson chi-square tests.

Accounting for the complex survey design (stratification, clustering, and weighting), three multivariate logistic regression models were constructed to evaluate the association between the RDW to albumin ratio (RAR) and the risk of hypertension: Model 1 (unadjusted); Model 2 adjusted for age, gender, and race/ethnicity; Model 3 additionally adjusted for socioeconomic status (poverty income ratio, education level, marital status), lifestyle factors (alcohol consumption, smoking status), and comorbidities (hypercholesterolemia, diabetes, kidney stones). Results are presented as odds ratios (OR) with 95% confidence intervals (CI).

Restricted cubic splines (RCS) were used to investigate the non-linear relationship between RAR and hypertension. Stratified analyses accounting for the complex survey design were performed along with interaction tests to evaluate potential effect modification across different subgroups.

## Results

### Baseline characteristics

In this nationally representative sample of U.S. adults (*N* = 7,878), significant gradients in sociodemographic and clinical characteristics were observed across increasing quartiles of RAR (all *P* < 0.05 except smoking, Table 1). Participants in higher RAR quartiles were older (Q1: 42.1 years vs. Q4: 52.9 years), more likely to be female (Q1: 38.5% vs. Q4: 70.9%), and had lower socioeconomic status (poverty income ratio: Q1: 3.26 vs. Q4: 2.62). A pronounced racial disparity was noted, with Non-Hispanic Black individuals comprising 4.8% of Q1 vs. 22.0% of Q4 (*P* < 0.001).Chronic disease burden escalated with RAR elevation: Hypertension prevalence increased from 29.6% (Q1) to 50.9% (Q4) (*P* < 0.001), diabetes from 6.6 to 18.9% (*P* < 0.001), and hypercholesterolemia from 30.0 to 37.3% (*P* = 0.003). Lifestyle factors showed modest associations, with alcohol consumption decreasing across quartiles (*P* = 0.016) but smoking prevalence remaining stable (*P* = 0.871). These findings confirm RAR as a biomarker strongly correlated with cardiometabolic risk profiles and underscore the necessity for multivariable adjustment in subsequent analyses.

**Table 1:** Weighted patient demographics and baseline characteristics

Quartiles of RAR Characteristic	Q1(*n* = 1961)	Q2(*n* = 1947)	Q3(*n* = 1994)	Q4(*n* = 1976)	*P*-value
Age (years)	42.08 (40.87,43.28)	48.96 (47.36,50.56)	52.87 (51.35,54.38)	52.86 (51.46,54.25)	< 0.0001
Ratio of family income to poverty	3.26 (3.12,3.39)	3.14 (3.01,3.27)	2.97 (2.85,3.08)	2.62 (2.45,2.78)	< 0.0001
Gender (%)					< 0.0001
Male	61.52 (57.65,65.24)	51.27 (48.59,53.93)	42.38 (38.85,45.98)	29.07 (26.28,32.03)	
Female	38.48 (34.76,42.35)	48.73 (46.07,51.41)	57.62 (54.02,61.15)	70.93 (67.97,73.72)	
Race/Ethnicity (%)					< 0.0001
Mexican American	8.48 (5.77,12.31)	8.46 (6.29,11.30)	8.29 (6.34,10.78)	8.87 (6.31,12.34)	
Other Hispanic	7.52 (5.77,9.74)	7.46 (5.80,9.54)	8.22 (6.55,10.27)	7.60 (5.64,10.16)	
Non-Hispanic White	68.42 (63.79,72.71)	66.84 (61.44,71.82)	60.52 (53.81,66.85)	52.69 (46.15,59.14)	
Non-Hispanic Black	4.75 (3.39,6.62)	7.45 (5.56,9.91)	13.66 (9.89,18.57)	22.02 (17.20,27.73)	
Other Race - Including Multi-Racial	10.83 (8.35,13.94)	9.80 (7.72,12.35)	9.31 (7.20,11.96)	8.82 (7.18,10.78)	
Education level (%)					< 0.0001
Less than college	33.13 (29.05,37.48)	36.11 (32.74,39.61)	40.28 (36.56,44.11)	45.91 (42.22,49.64)	
Some college or AA degree	27.63 (24.51,30.98)	33.07 (29.98,36.32)	31.32 (28.51,34.28)	30.92 (27.88,34.13)	
Some College graduate or above	39.25 (33.29,45.53)	30.82 (26.71,35.26)	28.40 (23.99,33.27)	23.17 (19.05,27.87)	
Marital status, n (%)					< 0.0001
Married/Living with Partner	62.87 (59.47,66.15)	64.90 (60.20,69.33)	61.05 (57.72,64.27)	58.13 (53.99,62.16)	
Widowed/Divorced/ Separated	12.80 (10.58,15.41)	17.02 (14.42,19.99)	23.90 (21.40,26.59)	24.57 (21.81,27.56)	
Never married	24.32 (21.78,27.06)	18.07 (14.53,22.26)	15.06 (12.72,17.73)	17.30 (14.21,20.89)	
Drink (%)					0.0156
Yes	95.20 (93.84,96.26)	94.07 (92.41,95.38)	92.52 (90.46,94.17)	92.42 (90.64,93.88)	
No	4.80 (3.74,6.16)	5.93 (4.62,7.59)	7.48 (5.83,9.54)	7.58 (6.12,9.36)	
hypercholesterolemia (%)					0.0027
Yes	29.96 (26.80,33.33)	35.74 (31.70,40.00)	37.82 (34.11,41.68)	37.27 (34.37,40.26)	
No	70.04 (66.67,73.20)	64.26 (60.00,68.30)	62.18 (58.32,65.89)	62.73 (59.74,65.63)	
Diabetes (%)					< 0.0001
Yes	6.62 (5.20,8.39)	10.28 (8.54,12.33)	14.05 (12.40,15.89)	18.89 (16.82,21.16)	
No	93.38 (91.61,94.80)	89.72 (87.67,91.46)	85.95 (84.11,87.60)	81.11 (78.84,83.18)	
Kidney stones (%)					0.0114
Yes	7.87 (6.23,9.90)	10.00 (8.59,11.63)	12.17 (9.61,15.31)	11.19 (8.65,14.36)	
No	92.13 (90.10,93.77)	90.00 (88.37,91.41)	87.83 (84.69,90.39)	88.81 (85.64,91.35)	
Smoking (%)					0.8706
Yes	42.79 (38.65,47.05)	41.89 (38.86,44.98)	42.66 (38.43,47.00)	44.26 (39.96,48.65)	
No	57.21 (52.95,61.35)	58.11 (55.02,61.14)	57.34 (53.00,61.57)	55.74 (51.35,60.04)	
Hypertension (%)					< 0.0001
Yes	29.57 (25.68,33.78)	37.54 (33.16,42.13)	45.29 (40.91,49.74)	50.88 (47.26,54.48)	
No	70.43 (66.22,74.32)	62.46 (57.87,66.84)	54.71 (50.26,59.09)	49.12 (45.52,52.74)	

Data in the table for continuous variables: survey-weighted mean (95% CI); For categorical variables: survey-weighted percentage (95% CI)

### The correlation between RAR and hypertension

In unadjusted analyses, elevated RAR levels exhibited a positive association with hypertension prevalence. Each unit increase in continuous RAR was associated with 85% higher odds of hypertension (OR = 1.85, 95% CI: 1.62–2.12, *P* < 0.001). Participants in the highest RAR quartile (Q4) had 2.47-fold higher odds compared to Q1 (95% CI: 2.00–3.04, *P* < 0.001), with a significant monotonic trend (P for trend < 0.001). After demographic adjustment (Model 2), the strength of association decreased but remained statistically significant for continuous RAR (OR = 1.41, 95% CI: 1.24–1.61) and Q4 (OR = 1.54, 95% CI: 1.26–1.88). Associations for Q2 and Q3 were no longer significant. In the fully adjusted model (Model 3), the highest RAR quartile maintained a statistically significant association with hypertension (OR = 1.29, 95% CI: 1.06–1.58, *P* = 0.013). Continuous RAR also showed remained associated (OR = 1.26, 95% CI: 1.09–1.44, *P* = 0.021), and the dose-response pattern remained significant (P for trend < 0.001). No significant associations were observed for intermediate quartiles (Q2–Q3). (Table 2).

**Table 2:** Association between RAR and hypertension risk

RAR Hypertension OR (95%CI)			
	Model 1(*n* = 7878) *P*	Model 2(*n* = 7878) *P*	Model 3 (*n* = 7878) *P*
RAR continues (*n* = 7878)	1.85 (1.62, 2.12) <0.001	1.41 (1.24, 1.61) <0.001	1.26 (1.09, 1.44) <0.05
Quartile			
Q1(*n* = 1961)	Reference	Reference	Reference
Q2(*n* = 1947)	1.43 (1.14, 1.80) <0.05	1.01 (0.77, 1.32) 0.958	0.95 (0.72, 1.26) 0.718
Q3(*n* = 1994)	1.97 (1.50, 2.60) <0.001	1.15 (0.88, 1.51) 0.300	1.04 (0.80, 1.35) 0.770
Q4(*n* = 1976)	2.47 (2.00, 3.04) <0.001	1.54 (1.26, 1.88) <0.001	1.29 (1.06, 1.58) <0.05
P for trend	< 0.001	< 0.001	< 0.001

*RAR* red cell distribution width-to-albumin ratio

Model 1: Unadjusted

Model 2: Adjusted for age, gender, race/ethnicity

Model 3: Adjusted for age, gender, race/ethnicity, poverty-income ratio, educational level, marital status, alcohol consumption, smoking, hypercholesterolemia, diabetes mellitus, kidney stones

Results are presented as Odds Ratio (OR) with 95% confidence interval (CI).

### Linear relationships between RAR and hypertension

**Fig. 2:**
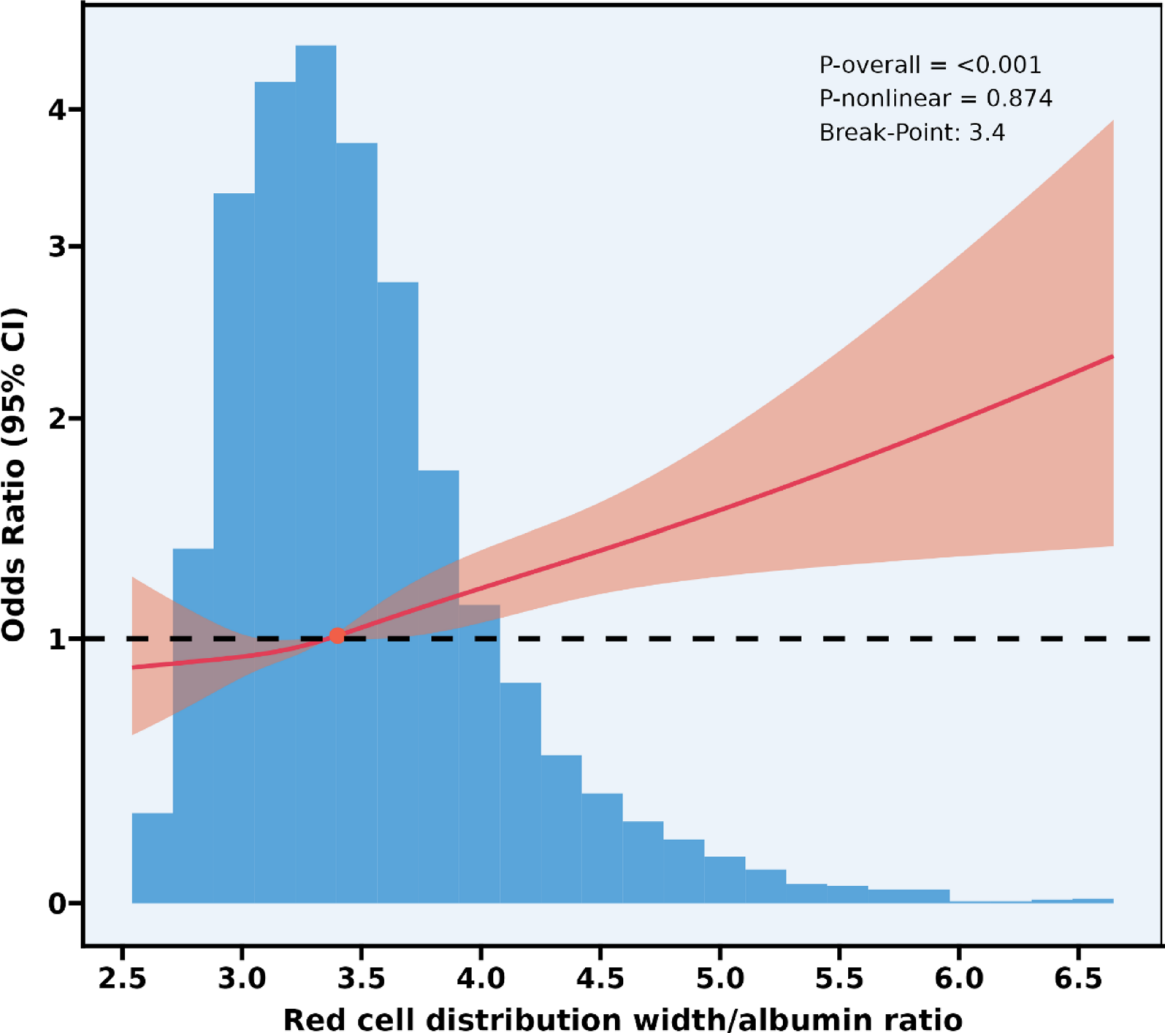
Restricted cubic splines for RAR and hypertension. Association between RAR and hypertension; A linear positive association was observed between RAR and hypertension risk (*p* < 0.001), after adjusting for age, gender, race/ethnicity, poverty-income ratio, educational level, marital status, alcohol consumption, smoking, hypercholesterolemia, diabetes mellitus, kidney stones. The solid line section represents the adjusted effect estimates, while the red shaded area delineates the 95% confidence interval. *RAR* red cell distribution width to albumin ratio.

Restricted cubic spline analysis revealed an approximately linear positive association between the red cell distribution width-to-albumin ratio (RAR) and hypertension prevalence (Overall *P* < 0.001; Nonlinearity *P* = 0.874) (Fig. 2), Despite a non-significant nonlinearty test (*P* = 0.874), the threshold model suggested distinct associations below and above RAR = 3.4, warranting clinical attention to this cutoff. The curve consistently resided above the reference line (OR = 1) when RAR ≥ 3.4. A two-part logistic regression model with a break-point at RAR = 3.4 demonstrated (Table 3): No significant association at RAR < 3.40 (OR = 1.14, 95% CI: 0.83–1.56, *P* = 0.408). A statistically significant positive association at RAR ≥ 3.40 (OR = 1.34, 95% CI: 1.17–1.54, *P* < 0.001). However, the log-likelihood ratio test indicated no significant improvement over the standard linear model (OR = 1.30, 95% CI: 1.17–1.44; *P* = 0.404).

**Table 3:** Threshold effect analysis of red cell distribution width-to-albumin ratio on hypertension

Models	Hypertension OR (95%CI)
Standard linear model	1.30 (1.17, 1.44) < 0.001
Two-part logistic regression model	
RAR < 3.40	1.14 (0.83, 1.56) 0.408
RAR ≥ 3.40	1.34 (1.17, 1.54) < 0.001
Log-likelihood ratio	0.404

*RAR* red cell distribution width-to-albumin ratio

Adjusted for age, gender, race/ethnicity, poverty-income ratio, educational level, marital status, alcohol consumption, smoking, hypercholesterolemia, diabetes mellitus, kidney stones.

### Subgroup analysis

Subgroup analyses revealed heterogeneity in the association between RAR exposure and hypertension risk. Significant positive associations were observed in females (OR = 1.30, 95%CI:1.12–1.50, *P* = 0.01), individuals aged 40–60 years (OR = 1.39, 95%CI:1.12–1.73, *P* = 0.03), and alcohol drinkers (OR = 1.24, 95%CI:1.07–1.42, *P* = 0.02). Subjects without kidney stones exhibited significantly elevated risk (OR = 1.30, *P* = 0.01). The most critical finding was a significant interaction with diabetes status (P for interaction = 0.02): diabetic patients demonstrated a 2.11-fold increased risk (95%CI:1.41–3.17, *P* = 0.01), substantially higher than non-diabetics (OR = 1.18, *P* = 0.08). No statistically significant associations were found in males (*P* = 0.21), those aged < 40 years (*P* = 0.45), hypercholesterolemia patients (OR = 1.40, 95%CI:0.98–2.02, *P* = 0.11), or kidney stone patients (*P* = 0.74). Although risk trends existed in other subgroups (age ≥ 60 years, non- hypercholesterolemia, smokers/non-smokers), no significant interactions were detected (P for interaction > 0.05) (see Table 4).

**Fig. 3:**
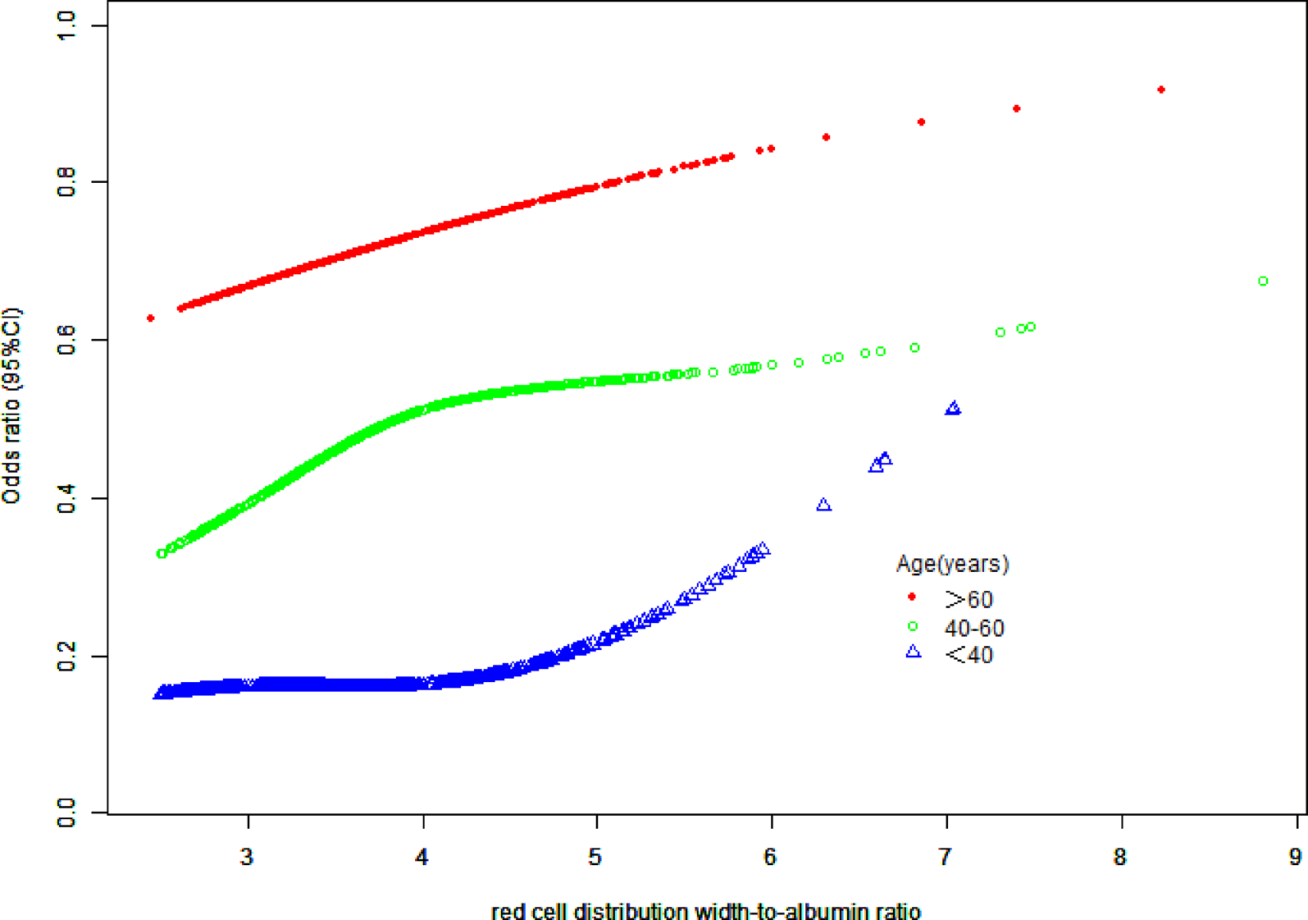
Smooth curve fitting diagram of RAR and hypertension by age stratification. Age-stratified threshold effect (40–60 years); Adjusted for gender, race, poverty-income ratio, educational attainment, marital status, alcohol consumption, smoking, hypercholesterolemia, diabetes mellitus, kidney stones

Notably, the age-stratified analysis (40–60 years) revealed a significant nonlinear relationship between RAR and hypertension risk( Fig. 3). Threshold effect analysis identified a critical inflection point at K = 3.92 (95%CI: 3.75–4.09): Below this threshold, each unit increase in RAR was associated with an 80% surge in hypertension risk (OR = 1.80, 95%CI = 1.33–2.43, *P* = 0.0001), whereas above this level, the risk trajectory plateaued without statistical significance (OR = 1.00, 95%CI = 0.76–1.32, *P* = 0.9975). The log-likelihood ratio test (*P* = 0.018) confirmed the statistical superiority of the nonlinear model(Table 5). Such a nonlinear relationship was not found among participants younger than 40 years old and those older than 60 years old.

**Table 4:** Subgroup analysis of the association between RAR and hypertension risk

Variable	*N*	OR (95%CI)	*P*	*P* for interaction	
Gender(%)				0.45	
Male	3807	1.17 (0.93,1.48)	0.21		
Female	4071	1.30 (1.12,1.50)	0.01		
Age				0.51	
< 40	2379	1.14 (0.83,1.57)	0.45		
40–60	2619	1.39 (1.12, 1.73)	0.03		
60+	2880	1.42 (1.03, 1.95)	0.08		
Hypercholesterolemia (%)				0.47	
Yes	2826	1.40 (0.98,2.02)	0.11		
No	5052	1.20 (1.03,1.40)	0.06		
Diabetes				0.02	
Yes	1219	2.11 (1.41,3.17)	0.01		
No	6659	1.18 (1.01,1.39)	0.08		
Kidney stones(%)				0.16	
Yes	751	0.93 (0.64,1.37)	0.74		
No	7127	1.30 (1.11,1.53)	0.01		
Smoking(%)				0.93	
Yes	3298	1.26 (1.03,1.55)	0.06		
No	4580	1.25 (1.06,1.47)	0.03		
Drink(%)				0.29	
Yes	7220	1.24 (1.07,1.42)	0.02		
No	658	1.68 (0.98,2.98)	0.1		

**Table 5:** Results of threshold effect analysis of RAR and hypertension by age stratification

Age(years)	40–60
Standard linear model	1.32 (1.12, 1.56) 0.0009
Two-part logistic regression model	
Inflection point (K)	3.92(3.76, 4.51)
RAR < 3.92	1.80 (1.33, 2.43) 0.0001
RAR ≥ 3.92	1.00 (0.76, 1.32) 0.9975
Log-likelihood ratio	0.018

## Discussion

This study is based on the cross-sectional data from the 2017 to 2020 National Health and Nutrition Examination Survey (NHANES) of the United States, which was conducted using complex sampling and weighted analysis. It was found that the red blood cell distribution width-to-albumin ratio (RAR) was significantly associated with the prevalence of hypertension, and this association exhibited a linear correlation feature. Further threshold analysis indicated that in the overall population, when RAR was ≥ 3.4, the prevalence of hypertension significantly increased (adjusted OR = 1.34, 95% CI: 1.17–1.54), while there was no statistical association when RAR was < 3.4 (*P* = 0.408). Age stratification analysis further revealed heterogeneity of the threshold: for the 40–60 age group, the turning point was at RAR = 3.92 (95% CI: 3.76–4.51), and the threshold-preceding interval (RAR < 3.92) showed a sharp increase in the prevalence of hypertension with the increase of RAR (OR = 1.80, 95% CI: 1.33–2.43), while the association disappeared after the threshold (*P* = 0.9975). Subgroup analysis indicated that this association had population heterogeneity: in diabetic patients, the association strength was significantly higher than that in non-diabetic patients (OR = 2.11 vs. 1.18, P-interaction = 0.02), and the association was more significant in the 40 + age group and the female subgroup (such as the 40–60 age group, OR = 1.39, *P* = 0.03). These results suggest that RAR, as a composite indicator reflecting inflammation and nutritional status, may become an effective marker for identifying high-risk subgroups of hypertension, especially in diabetic and middle-aged populations.

Hypertension is the leading non-infectious cause of death, greatly surpassing other significant factors contributing to mortality, such as smoking and metabolic diseases [25, 26]. It is a major risk factor for myocardial infarction, stroke, kidney damage, and cognitive impairment [27]. Nearly 1.5 billion people worldwide suffer from hypertension, with the vast majority having essential hypertension and a small portion having secondary hypertension. The pandemic nature of the prevalence of hypertension has imposed a huge health and socioeconomic burden worldwide [28]. Inflammation plays an important role in the pathophysiology of hypertension [29, 30]. Chronic low-grade inflammation can drive up blood pressure by promoting vascular endothelial dysfunction [3, 31], oxidative stress [32], and the activation of the renin-angiotensin-aldosterone system (RAAS) [33, 34]. 

As a novel comprehensive inflammatory biomarker, the red cell distribution width-to-albumin ratio (RAR) has been proven to be involved in the occurrence and development of various inflammatory diseases, such as diabetes mellitus, stroke, and chronic kidney disease [35–37]. Our analysis showed that there was an independent positive correlation between RAR and hypertension. Each 1-unit higher RAR was associated with 26% (OR = 1.26) greater hypertension prevalence. The association between RAR and hypertension may involve its dual regulation of systemic inflammation and oxidative stress [38]. First, elevated RDW may reflect abnormal erythropoiesis, chronic inflammatory states, and oxidative stress. Inflammatory factors (such as TNF-α, IL-6) inhibit erythrocyte maturation, leading to increased heterogeneity in red blood cell size in circulation [39]. A decrease in albumin levels indicates systemic inflammation, malnutrition, or liver and kidney dysfunction [40]. Hypoalbuminemia weakens plasma antioxidant capacity, reduces nitric oxide (NO) bioavailability, and directly promotes vascular endothelial dysfunction [41], thereby increasing peripheral resistance [42]. Second, it may be related to RAAS activation. Inflammatory factors stimulate the renin-angiotensin-aldosterone system (RAAS), increasing the production of angiotensin II, promoting vascular smooth muscle proliferation and vasoconstriction, increasing peripheral resistance, and thus exacerbating vasoconstriction and sodium retention [43–46]. Finally, by integrating RDW and albumin, RAR directly reflects the severity of the above pathological processes [38]. Therefore, The association between RAR and hypertension may reflect continuous inflammation activation. which is implicated in hypertension pathogenesis.

The subgroup heterogeneity discovered in this study provided important clues for precise prevention and control. The strength of association between RAR and hypertension was 1.87 times greater in diabetics than non-diabetics. This may be related to the continuous activation of cytokines such as interleukin-1 (IL-1), interleukin-6 (IL-6), and tumor necrosis factor-α (TNF-α) in diabetic patients [47], and the fact that hyperglycemia is associated with ROS production, leading to oxidative stress [48]. Meanwhile, oxidized low-density lipoprotein (ox-LDL) and the inflammatory pathways related to RAR may synergistically promote monocyte adhesion and pro-inflammatory activation of endothelial cells through the LOX-1 receptor signaling, thereby exacerbating vascular dysfunction [49]. 

The nonlinear association observed in the 40–60 age group (with an inflection point at RAR = 3.92) carries significant clinical implications. When RAR exceeds 3.92, the magnitude of increased hypertension risk appears to diminish. This finding suggests that in middle-aged adults, RAR levels below 3.92 showed a steeper increase in hypertension prevalence compared to higher levels: cross-sectional data indicate an association between RAR levels below 3.92 and slower progression of hypertension. Potential mechanisms may involve age-related decline in vascular elasticity, cumulative oxidative stress, significant deterioration of compensatory capacity, and the synergistic amplification of vascular damage by metabolic syndrome (diabetes, hypercholesterolemia) and RAR [50, 51]. Large-sample prospective clinical studies are needed to further explore the underlying mechanisms and potential significance of this association.

Although this study first revealed the independent association between RAR and hypertension, it also has some limitations. Firstly, since this is a cross-sectional analysis, it is impossible to determine the causal relationship. Secondly, self-reported hypertension may be biased because it stems from those hypertensive patients who did not seek medical treatment and did not have their blood pressure measured, as well as those whose blood pressure was not measured. Moreover, confounding factors such as C-reactive protein levels and medication status were not considered. The cross-sectional design of this study cannot clarify the temporal sequence between RAR and hypertension, and there is a risk of reverse causality bias. Although this study provides valuable epidemiological insights, its direct clinical significance may be limited. Therefore, more rigorous prospective cohort studies or randomized controlled trials are needed in the future to determine the causal relationship between RAR and hypertension, reduce the interference of confounding factors, and further verify the reliability of the results of this study.

Finally, as an indicator that comprehensively considers inflammation and metabolic imbalance, RAR is a novel biomarker associated with hypertension status. In the framework of precision medicine, by combining age-specific thresholds (such as a RAR value of 3.92 for middle-aged people) and metabolic comorbidity status to provide reference indicators for risk stratification, it may bring new insights to the assessment of hypertension risk.

## Conclusion

Based on the cross-sectional data of the National Health and Nutrition Examination Survey (NHANES) in the United States from 2017 to 2020 obtained through complex sampling weighted analysis, this study confirmed a linear positive correlation between red blood cell distribution width and albumin ratio (RAR) and the prevalence of hypertension. Although the non-linear test results were not significant (*P* = 0.874), the threshold model clearly distinguished the risk differences between RAR < 3.4 and ≥ 3.4 situations. This might have clinical value in risk stratification as the higher range shows a statistically significant association with hypertension. In the general population, RAR ≥ 3.4 was significantly positively correlated with the prevalence of hypertension (weighted adjusted OR value was 1.34, 95% confidence interval was 1.17–1.54), while there was no statistical significance when RAR < 3.4 (*P* = 0.408); for the 40–60 age group, there was a unique threshold response, with the inflection point located at RAR = 3.92 (95% confidence interval was 3.76–4.51). In the interval before the threshold (RAR < 3.92), the association strength significantly increased (weighted OR value was 1.80, 95% confidence interval was 1.33–2.43). Group analysis indicated that this association showed population heterogeneity: the association strength was significantly higher in diabetic patients than in non-diabetic patients (interaction *P* = 0.02), and the association was more significant in the age group above 40 years and the female subgroup. As a routine blood indicator, the threshold effect (≥ 3.4) of RAR (a new biomarker related to hypertension status) is a novel biomarker, but its predictive effect needs to be verified through prospective cohort studies.

## Data Availability

The datasets analyzed in this study are publicly available from the National Health and Nutrition Examination Survey (NHANES) 2017-2020 cycle. All data can be accessed through the official NHANES website at: https://wwwn.cdc.gov/nchs/nhanes/Default.aspx. Researchers must register and agree to the data use agreement prior to downloading the data.

## References

[1] Wang L, Song TT, Dong CW. Association between interactions among ACE gene polymorphisms and essential hypertension in patients in the Hefei region, anhui, China. J Renin Angiotensin Aldosterone Syst. 2023;2023:1159973. 10.1155/2023/1159973. 37091860 10.1155/2023/1159973 PMC10118893

[2] Gentile G, Mckinney K, Reboldi G. Tight blood pressure control in chronic kidney disease. J Cardiovasc Dev Dis. 2022;9(5):139. 10.3390/jcdd9050139. 35621850 10.3390/jcdd9050139 PMC9144041

[3] Hu S, Zhang Y, Qiu C, Li Y. RGS10 inhibits proliferation and migration of pulmonary arterial smooth muscle cell in pulmonary hypertension via AKT/mTORC1 signaling. Clin Exp Hypertens. 2023;45(1):2271186. 10.1080/10641963.2023.2271186. 37879890 10.1080/10641963.2023.2271186

[4] Kandasamy G, Subramani T, Sam G, et al. Biosocial predictors and blood pressure goal attainment among postmenopausal women with hypertension. Front Cardiovasc Med. 2024;11:1268791. 10.3389/fcvm.2024.1268791. 38433758 10.3389/fcvm.2024.1268791 PMC10906718

[5] ZZ. Role of inflammation, immunity, and oxidative stress in hypertension: new insights and potential therapeutic targets. Front Immunol. 2023;13. 10.3389/fimmu.2022.1098725. 10.3389/fimmu.2022.1098725 PMC9871625 36703963

[6] CBB, FlVC. Skin autofluorescence predicts major adverse cardiovascular events in patients with type 1 diabetes: a 7-year follow-up study. Cardiovasc Diabetol. 2018;17(1). 10.1186/s12933-018-0718-8. 10.1186/s12933-018-0718-8 PMC5993997 29884175

[7] Huang M, Liu F, Li Z, et al. Relationship between red cell distribution width/albumin ratio and carotid plaque in different glucose metabolic States in patients with coronary heart disease: a RCSCD-TCM study in China. Cardiovasc Diabetol. 2023;22(1):39. 10.1186/s12933-023-01768-w. 36814226 10.1186/s12933-023-01768-w PMC9948352

[8] Sa O, Ar A. The effect of red cell distribution width admission value on the outcome of patients with First-ever ST-elevation myocardial infarction in Basrah. Cureus. 2020;12(3). 10.7759/cureus.7373. 10.7759/cureus.7373 PMC7176332 32328384

[9] de Paula HL, de Lucca L, Vendrame SA, et al. Delta-aminolevulinate dehydratase enzyme activity and the oxidative profile of pregnant women being treated for acute toxoplasmosis. Microb Pathog. 2022;164:105455. 10.1016/j.micpath.2022.105455. 35219844 10.1016/j.micpath.2022.105455

[10] Marzouk H, Mostafa N, Khalifa I, Badawi N, Mohamed Fathy Sabry NI. Red cell distribution width (RDW) as a marker of subclinical inflammation in children with Familial mediterranean fever. Curr Rheumatol Rev. 2020;16(4):298–303. 10.2174/1573397116666200312142709. 32164513 10.2174/1573397116666200312142709

[11] Saitou T, Watanabe K, Kinoshita H, et al. Hypoalbuminemia is related to endothelial dysfunction resulting from oxidative stress in parturients with preeclampsia. Nagoya J Med Sci. 2021;83(4):741–8. 10.18999/nagjms.83.4.741. 34916718 10.18999/nagjms.83.4.741 PMC8648536

[12] Watanabe K, Kinoshita H, Okamoto T, Sugiura K, Kawashima S, Kimura T. Antioxidant properties of albumin and diseases related to obstetrics and gynecology. Antioxid (Basel). 2025;14(1):55. 10.3390/antiox14010055. 10.3390/antiox14010055 PMC11760856 39857389

[13] Ding J, Zhang Y, Chen X. Red cell distribution width to albumin ratio is associated with asthma risk: a population-based study. Front Med (Lausanne). 2024;11:1493463. 10.3389/fmed.2024.1493463. 39722824 10.3389/fmed.2024.1493463 PMC11668568

[14] Chen S, Guan S, Yan Z, et al. Prognostic value of red blood cell distribution width-to-albumin ratio in ICU patients with coronary heart disease and diabetes mellitus. Front Endocrinol (Lausanne). 2024;15:1359345. 10.3389/fendo.2024.1359345. 39387054 10.3389/fendo.2024.1359345 PMC11461254

[15] A DL. Association between red blood cell distribution width-to-albumin ratio and the prognosis in patients with autoimmune encephalitis: a retrospective cohort study. Front Neurol. 2024;14. 10.3389/fneur.2023.1276026. 10.3389/fneur.2023.1276026 PMC10808499 38274889

[16] Zhang C, Lu S, Kang T, et al. Red cell distribution width/albumin ratio and mortality risk in rheumatoid arthritis patients: insights from a NHANES study. Int J Rheum Dis. 2024;27(9):e15335. 10.1111/1756-185X.15335. 39278721 10.1111/1756-185X.15335

[17] Lan C, Fang G, Qiu C, Li X, Yang F, Yang Y. Inhibition of DYRK1A attenuates vascular remodeling in pulmonary arterial hypertension via suppressing STAT3/Pim-1/NFAT pathway. Clin Exp Hypertens. 2024;46(1):2297642. 10.1080/10641963.2023.2297642. 38147409 10.1080/10641963.2023.2297642

[18] Ma C, Liang G, Wang B, et al. Clinical value of the red blood cell distribution width to albumin ratio in the assessment of prognosis in critically ill patients with sepsis: a retrospective analysis. J Thorac Dis. 2024;16(1):516–29. 10.21037/jtd-23-1696. 38410549 10.21037/jtd-23-1696 PMC10894361

[19] Tan Y, Li Y, Huang X, et al. The ratio of red blood cell distribution width to albumin as a predictor for rehospitalization risk and rehospitalization All-Cause mortality in Middle-Aged and elderly survivors with sepsis: an ambispective ICU cohort study. J Inflamm Res. 2024;17:1227–40. 10.2147/JIR.S451769. 38410420 10.2147/JIR.S451769 PMC10896106

[20] Liu R, Zhong L, Wang C, et al. MiR-3646 accelerates inflammatory response of Ang II-induced hVSMCs via CYP2J2/EETs axis in hypertension model. Clin Exp Hypertens. 2023;45(1):2166948. 10.1080/10641963.2023.2166948. 36751048 10.1080/10641963.2023.2166948

[21] Zhou P, Tian PC, Zhai M, et al. Association between red blood cell distribution width-to-albumin ratio and prognosis in non-ischaemic heart failure. ESC Heart Fail. 2024;11(2):1110–20. 10.1002/ehf2.14628. 38266632 10.1002/ehf2.14628 PMC10966226

[22] Lin M, Wang X, Ye B, et al. External counterpulsation stimulation combined with acupuncture for vascular endothelial function in patients with hypertension: A randomized pilot trial. Clin Exp Hypertens. 2023;45(1):2181355. 10.1080/10641963.2023.2181355. 36871563 10.1080/10641963.2023.2181355

[23] Celik SU, Gulap Y, Demir MB, Demircioglu MM, Polat HE, Kesikli SA. Predictive value of the red cell distribution Width-To-Albumin ratio for clinical outcomes in patients with peptic ulcer perforation. World J Surg Published Online Febr. 2025;24. 10.1002/wjs.12515. 10.1002/wjs.12515 PMC11994152 39993970

[24] Zhao L, Zheng L, Wang R, et al. Association between triglyceride glucose combined with body mass index and hypertension in the NHANES 2017 to 2020. Sci Rep. 2025;15:9092. 10.1038/s41598-025-93723-w. 40097561 10.1038/s41598-025-93723-w PMC11914623

[25] Dong L, Liu J, Qin Y, et al. Relationship between ambulatory arterial stiffness index and the severity of angiographic atherosclerosis in patients with H-type hypertension and coronary artery disease. Clin Exp Hypertens. 2023;45(1):2228517. 10.1080/10641963.2023.2228517. 37358029 10.1080/10641963.2023.2228517

[26] Isordia-Salas I, Santiago-Germán D, Jiménez-Alvarado RM, Leaños-Miranda A. Genetic variants associated with high susceptibility of premature ischemic stroke. J Renin Angiotensin Aldosterone Syst. 2023;2023:9002021. 10.1155/2023/9002021. 38025202 10.1155/2023/9002021 PMC10667057

[27] Deussen A, Kopaliani I. Targeting inflammation in hypertension. Curr Opin Nephrol Hypertens. 2023;32(2):111–7. 10.1097/MNH.0000000000000862. 36476561 10.1097/MNH.0000000000000862 PMC9872860

[28] Deng X, Luo H, He J, Deng W, Wang D. Omentin-1 ameliorates pulmonary arterial hypertension by inhibiting Endoplasmic reticulum stress through AMPKα signaling. Clin Exp Hypertens. 2024;46(1):2332695. 10.1080/10641963.2024.2332695. 38527024 10.1080/10641963.2024.2332695

[29] Dai C, Tan M, Meng X, Dong J, Zhang Y. Effects of potassium channel knockdown on peripheral blood T lymphocytes and NFAT signaling pathway in Xinjiang Kazak patients with hypertension. Clin Exp Hypertens. 2023;45(1):2169449. 10.1080/10641963.2023.2169449. 36691302 10.1080/10641963.2023.2169449

[30] Dong J, Jiang XM, Xie DJ, et al. Establishment of a canine model of pulmonary arterial hypertension induced by dehydromonocrotaline and ultrasonographic study of right ventricular remodeling. Clin Exp Hypertens. 2023;45(1):2190503. 10.1080/10641963.2023.2190503. 36924239 10.1080/10641963.2023.2190503

[31] Ritson M, Wheeler-Jones CPD, Stolp HB. Endothelial dysfunction in neurodegenerative disease: is endothelial inflammation an overlooked druggable target? J Neuroimmunol. 2024;391:578363. 10.1016/j.jneuroim.2024.578363. 38728929 10.1016/j.jneuroim.2024.578363

[32] Weinberg Sibony R, Segev O, Dor S, Raz I. Overview of oxidative stress and inflammation in diabetes. J Diabetes. 2024;16(10):e70014. 10.1111/1753-0407.70014. 39435991 10.1111/1753-0407.70014 PMC11494684

[33] Pillai A, Fulmali D. A narrative review of new treatment options for diabetic nephropathy. Cureus. 2023;15(1):e33235. 10.7759/cureus.33235. 36733548 10.7759/cureus.33235 PMC9889842

[34] Schieffer E, Schieffer B. The race for ACE: targeting Angiotensin-Converting enzymes (ACE) in SARS-CoV-2 infection. J Renin Angiotensin Aldosterone Syst. 2022;2022:2549063. 10.1155/2022/2549063. 35685188 10.1155/2022/2549063 PMC9166989

[35] Li D, Long J, Zhang J, et al. Association between red cell distribution width-and-albumin ratio and the risk of peripheral artery disease in patients with diabetes. Front Endocrinol (Lausanne). 2024;15:1272573. 10.3389/fendo.2024.1272573. 38405142 10.3389/fendo.2024.1272573 PMC10884210

[36] Xu Y, Qi W. Association between red cell distribution width to albumin ratio and acute kidney injury in patients with sepsis: a MIMIC population-based study. Int Urol Nephrol. 2023;55(11):2943–50. 10.1007/s11255-023-03572-7. 37014490 10.1007/s11255-023-03572-7

[37] Zhao J, Feng J, Ma Q, Li C, Qiu F. Prognostic value of inflammation biomarkers for 30-day mortality in critically ill patients with stroke. Front Neurol. 2023;14:1110347. 10.3389/fneur.2023.1110347. 36814998 10.3389/fneur.2023.1110347 PMC9939760

[38] Aslam H, Oza F, Ahmed K, et al. The role of red cell distribution width as a prognostic marker in chronic liver disease: A literature review. Int J Mol Sci. 2023;24(4):3487. 10.3390/ijms24043487. 36834895 10.3390/ijms24043487 PMC9967940

[39] Ding J, Chen J, Zhou J, Jiang Z, Xiang D, Xing W. Association between renal surface nodularity and increased adverse vascular event risk in patients with arterial hypertension. Clin Exp Hypertens. 2023;45(1):2228518. 10.1080/10641963.2023.2228518. 37366048 10.1080/10641963.2023.2228518

[40] Jiang D, Matsuzaki M, Kawagoe Y, et al. Analysis of mechanisms for increased blood pressure variability in rats continuously infused with angiotensin II. J Renin Angiotensin Aldosterone Syst. 2023;2023:4201342. 10.1155/2023/4201342. 36704758 10.1155/2023/4201342 PMC9833913

[41] Li X, Zhang X, Zeng Z, et al. Serum albumin and prognosis in elderly patients with nonischemic dilated cardiomyopathy. J Cardiovasc Med (Hagerstown). 2023;24(10):752–7. 10.2459/JCM.0000000000001530. 37577864 10.2459/JCM.0000000000001530 PMC10481926

[42] Verbeke F, Vanholder R, Van Biesen W, Glorieux G. Contribution of hypoalbuminemia and Anemia to the prognostic value of plasma p-Cresyl sulfate and p-Cresyl glucuronide for cardiovascular outcome in chronic kidney disease. J Pers Med. 2022;12(8):1239. 10.3390/jpm12081239. 36013188 10.3390/jpm12081239 PMC9410048

[43] Choopani S, Nematbakhsh M. Estradiol supplement or induced hypertension May attenuate the angiotensin II type 1 receptor Antagonist-Promoted renal blood flow response to graded angiotensin II administration in ovariectomized rats. J Renin Angiotensin Aldosterone Syst. 2022;2022:3223008. 10.1155/2022/3223008. 35859805 10.1155/2022/3223008 PMC9270140

[44] Murasawa S, Kageyama K, Usutani M, et al. Biochemical evaluation by confirmatory tests after unilateral adrenalectomy for primary aldosteronism. J Renin Angiotensin Aldosterone Syst. 2023;2023:5732812. 10.1155/2023/5732812. 37265473 10.1155/2023/5732812 PMC10232090

[45] Vg F, Bm GL. Nephrotic syndrome: from pathophysiology to novel therapeutic approaches. Biomedicines. 2024;12(3). 10.3390/biomedicines12030569. 10.3390/biomedicines12030569 PMC10968602 38540182

[46] Zhang N, Huo Y, Yao C, Sun J, Zhang Y. The effect of the Angiotensin-Converting enzyme inhibitor on bone health in castrated hypertensive rats is mediated via the Kinin-Kallikrein system. J Renin Angiotensin Aldosterone Syst. 2022;2022:9067167. 10.1155/2022/9067167. 35814865 10.1155/2022/9067167 PMC9213206

[47] Rohm TV, Meier DT, Olefsky JM, Donath MY. Inflammation in obesity, diabetes, and related disorders. Immunity. 2022;55(1):31–55. 10.1016/j.immuni.2021.12.013. 35021057 10.1016/j.immuni.2021.12.013 PMC8773457

[48] L MY. A novel inflammatory marker: relationship between red cell distribution width/albumin ratio and vascular complications in patients with type 2 diabetes mellitus. J Inflamm Res. 2024;17. 10.2147/JIR.S476048. 10.2147/JIR.S476048 PMC11401529 39281773

[49] Tuz AA, Hoerenbaum N, Ulusoy Ö, et al. Hypercholesterolemia triggers innate immune imbalance and transforms brain infarcts after ischemic stroke. Front Immunol. 2024;15:1502346. 10.3389/fimmu.2024.1502346. 39845972 10.3389/fimmu.2024.1502346 PMC11750678

[50] Guo X, Guo Y, Wang Z, et al. Reducing the damage of Ox-LDL/LOX-1 pathway to vascular endothelial barrier can inhibit atherosclerosis. Oxid Med Cell Longev. 2022;2022:7541411. 10.1155/2022/7541411. 35391927 10.1155/2022/7541411 PMC8983252

[51] Isordia-Salas I, Santiago-Germán D, Flores-Arizmendi A, Leaños-Miranda A. Polymorphisms in the Renin-Angiotensin system and eNOS Glu298Asp genes are associated with increased risk for essential hypertension in a Mexican population. J Renin Angiotensin Aldosterone Syst. 2023;2023:4944238. 10.1155/2023/4944238. 36845669 10.1155/2023/4944238 PMC9957645

